# Imaging cAMP nanodomains in the heart

**DOI:** 10.1042/BST20190245

**Published:** 2019-10-31

**Authors:** Ying-Chi Chao, Nicoletta C. Surdo, Sergio Pantano, Manuela Zaccolo

**Affiliations:** 1 Department of Physiology, Anatomy and Genetics, University of Oxford, Oxford, U.K.; 2 Institut Pasteur de Montevideo, Montevideo, Uruguay

**Keywords:** cAMP, compartmentalization, FRET imaging, nanodomain

## Abstract

3′-5′-cyclic adenosine monophosphate (cAMP) is a ubiquitous second messenger that modulates multiple cellular functions. It is now well established that cAMP can mediate a plethora of functional effects via a complex system of local regulatory mechanisms that result in compartmentalized signalling. The use of fluorescent probes to monitor cAMP in intact, living cells have been instrumental in furthering our appreciation of this ancestral and ubiquitous pathway and unexpected details of the nano-architecture of the cAMP signalling network are starting to emerge. Recent evidence shows that sympathetic control of cardiac contraction and relaxation is achieved via generation of multiple, distinct pools of cAMP that lead to differential phosphorylation of target proteins localized only tens of nanometres apart. The specific local control at these nanodomains is enabled by a distinct signalosome where effectors, targets, and regulators of the cAMP signal are clustered. In this review, we focus on recent advances using targeted fluorescent reporters for cAMP and how they have contributed to our current understanding of nanodomain cAMP signalling in the heart. We briefly discuss how this information can be exploited to design novel therapies and we highlight some of the questions that remain unanswered.

## Introduction

The second messenger 3′,5′-cyclic adenosine monophosphate (cAMP) is involved in multiple physiological functions. In response to a variety of different hormones and other extracellular cues that operate via activation of G protein-coupled receptors (GPCRs), cAMP affects cellular processes that range from modulation of metabolic enzymes to regulation of transcription, from the control of cell survival to cross-talk with multiple other signalling pathways. In eukaryotic cells, cAMP activates three ubiquitously expressed intracellular cAMP effector proteins, the cAMP-dependent protein kinase A (PKA), the cyclic nucleotide gated ion channels, and the exchange protein activated by cAMP (Epac) [1]. The presence of these three cAMP effector families contributes to convey a precise and integrated functional outcome to the cAMP signals. A fourth family of proteins, the Popeye domain containing proteins bind cAMP, although their specific function remains unclear [2].

In the heart, cAMP signalling mediates the sympathetic fight-or-flight response by regulating heart rate and strength of contraction via modulation of several aspects of the excitation–contraction coupling (ECC) process. ECC involves activation of β-adrenergic receptors (β-ARs) by catecholamines followed by synthesis of cAMP, activation of PKA and phosphorylation of a numbers of key protein targets. Direct activation of I_f_ channels by cAMP and PKA-dependent phosphorylation of L-type Ca^2+^ channels (LTCC) at the sarcolemma (SL) and phosphorylation of ryanodine receptors (RyRs) and phospholamban (PLB) at the sarcoplasmic reticulum (SR) results in increased amplitude of the Ca^2+^ transient and enhanced heart rate (chronotropy) and strength of contraction (inotropy); by promoting Ca^2+^ reuptake into the SR, PKA-dependent phosphorylation of PLB also enhances relaxation (lusitropy); at the myofilaments (MF), PKA phosphorylation of troponin I (TPNI) reduces MF Ca^2+^ sensitivity and phosphorylation of myosin-binding protein C (MyBPC) increases the speed of cross-bridge formation, resulting in enhanced cardiac relaxation and contraction, respectively [3]. As this complex β-AR-dependent regulation of ECC takes place, cAMP carries out a myriad of other signalling functions that are triggered by different hormones and receptors.

Understanding how cardiac myocytes respond specifically to different stimuli that signal via cAMP and PKA has been the focus of intense investigations in recent years. The current view is that regulation of this intricate signalling system relies on compartmentalization of cAMP at the nanoscale level, a process that results in the tailored local concentration of the second messenger to achieve selective activation of downstream targets. The development of fluorescent biosensors for real-time detection of cAMP in intact cells has been instrumental in unravelling the compartmentalized nature of cAMP signalling and recently developed targeted reporters are providing key information on the organization and regulation of cAMP subcellular nanodomains [4]. A cAMP nanodomain is a subcellular site of nanometres dimension within which the level of cAMP is different from the surrounding environment. Typically a multiprotein complex, or signalosome, including a cAMP effector and a target is contained within the nanodomain and is regulated by the specific local concentration of cAMP. This knowledge is changing our understanding of cardiac myocyte physiology and provides a novel framework for the development of more effective, targeted therapies.

## cAMP and nanodomains

Research in the last two decades has clearly established that compartmentalization plays a key role in determining the specificity of cAMP/PKA signalling. This was first hypothesized on the basis of the observation that despite generating similar elevation of global cAMP levels, catecholamines mainly activate type II isoforms of PKA and promote cardiac inotropy and lusitropy, whereas prostaglandins activate type I PKA and have no effect on cardiac muscles contraction [5]. To explain these observations, the hypothesis was formulated that, on activation, β-ARs and prostaglandin receptors engage spatially distinct subcellular domains, leading to the activation of a selected subset of effectors and phosphorylation of distinct targets.

There is now ample evidence to support this model. Studies have shown that compartmentalized cAMP/PKA signalling is achieved via spatial segregation of the molecular components that carry out these signalling events into distinct signalosomes [6]. Typically, within a signalosome, an A-kinase anchoring protein (AKAP) tethers PKA in proximity to a specific phosphorylation target [7]. Selective activation of these anchored PKA subsets is then achieved by confining the increase in cAMP triggered by a specific stimulus to the subcellular site within which the relevant AKAP/PKA/target signalosome resides, enabling rapid and selective PKA-dependent phosphorylation [8–10]. Signalosomes often include adenylyl cyclases (AC) that generate cAMP, phosphodiesterases (PDEs), a superfamily of enzymes comprising multiple isoforms that degrade cAMP, and phosphatases (PPs), that dephosphorylate PKA targets.

In ventricular myocytes, several AKAP complexes have been identified in different subcellular domains. For example, Yotiao, also known as AKAP9, forms a complex with AC2/9, the potassium channel subunit KCNQ1 and the phosphatase PP1 at the SL and this complex is necessary for cAMP-dependent regulation of K^+^ current [11]; the AKAP79/β-AR/AC/LTCC complex also localizes at the SL where local activation of PKA regulates the Ca^2+^ influx through LTCC [12–14]; the AKAP18δ/SERCA/PLB/PDE3A complex at the SR regulates Ca^2+^ reuptake via selective phosphorylation of PLB [15,16]; an mAKAP/RyR2/PDE4D3 complex has been found at the nuclear envelop where it regulates RyR phosphorylation and multiple other signalling functions [17]; at the MF, troponin T [18], α-synemin [19], and myosprin [20] have been proposed to act as AKAPs, although less information is available on the composition of these potential signalosomes. A significant body of work has demonstrated that PDEs, by hydrolyzing cAMP at specific cellular sites, play a key role in shaping cAMP nanodomains and a detailed account of these investigations can be found in many recent reviews [21,22].

## Targeted FRET-based sensors for cAMP

Development of fluorescence resonance energy transfer (FRET)-based reporters has made it possible to directly image compartmentalized cAMP in living cells through direct visualization of local changes in cAMP levels. The possibility to monitor cAMP signalling as it happens in intact, living cells, where the complexity of the intracellular architecture and the spatial organization of the biochemical machinery is preserved, has been instrumental in defining the compartmentalized nature of cAMP signalling and is starting to provide unexpected, novel insight into cell physiology. Targeting these sensors to specific subcellular locations is proving particularly fruitful.

**Figure 1.**
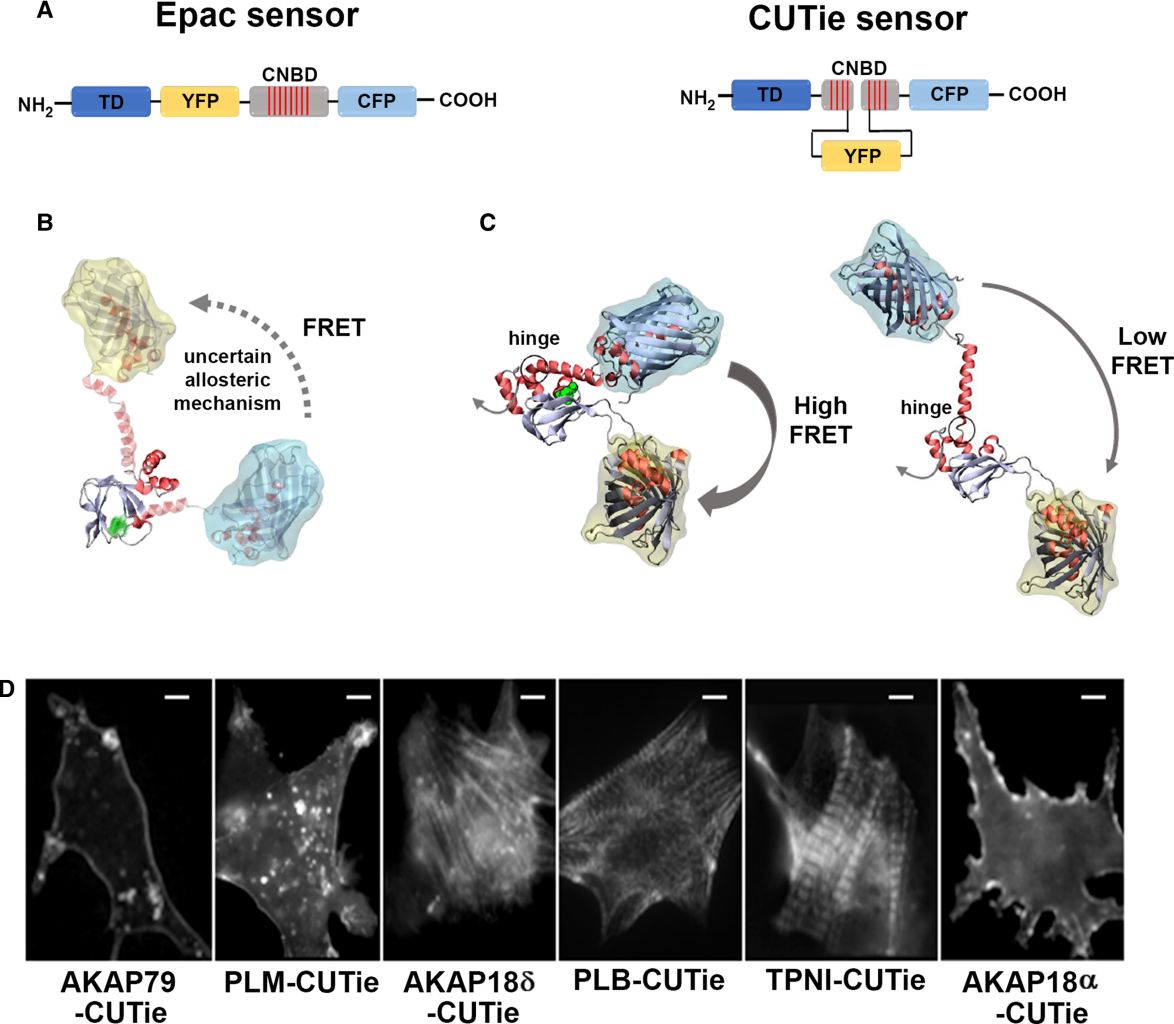
Molecular insights into Epac and CUTie sensors. (**A**) Schematic representation of the targeted Epac and CUTie sensors. CNBD, cyclic nucleotide-binding domain; TD, targeting domain. (**B**) Cartoon model of the Epac sensor constructed using the Apo structure of Epac2 [49]. The model is coloured by secondary structure (red: helix, grey: coil, light blue: extended beta) and cAMP is green. The helical regions at the amino- and carboxy-termini of the CNBD are shown in semi-transparent materials to highlight that the allosteric mechanism responsible for the increase in FRET on cAMP release is uncertain (see text for details). The surfaces around the florescent modules are coloured yellow and cyan fort YFP and CFP, respectively. (**C**) The CUTie sensor in its cAMP-bound and free conformations, respectively. Models are taken from simulations presented in [9] and coloured as in (**B**). The grey arrow at the amino-terminus indicates the site where the sensor can be fused to the carboxy-terminus of targeting proteins. The black circle marks the hinge position. (**D**) Neonatal cardiac myocytes expressing targeted CUTie reporters. The identity of the targeting domain is indicated in the prefix. Scale bar: 10 µm. PLM, phospholemman; PLB, phospholamban; TPNI, troponin I.

A successful family of FRET-based reporter used the cAMP effector protein Epac or its cyclic nucleotide-binding domain (CNBD) sandwiched between yellow fluorescent protein and cyan fluorescent protein (YFP–CFP) [23–25]. The so-called Epac sensor was developed making rough estimations of suitable insertion points for yellow and cyan fluorescent modules based on the structural information available at the time for the Epac2 protein [24]. This sensor develops a strong decrease in FRET upon increasing concentrations of cAMP. Using the structure of Epac2 as structural template to model the CNBD in the presence of both fluorescent modules (Figure 1B) shows a couple of features that make it difficult to completely rationalize the molecular basis for the allosteric mechanism. Firstly, in the model, a long (putatively) helical segment connects the FRET acceptor to the CNBD. The integrity of this helical segment in the absence of its entire tertiary context is unlikely. Secondly, the insertion of the FRET donor truncates the cAMP pocket, eliminating the lid that eventually closes onto the second messenger. Although a detailed structural rationalization is, indeed, not required for the practical use of the sensor as a cytosolic probe, this architecture generates a major drawback when the sensor is targeted to specific cellular subdomains. Since the amino- and carboxy-termini correspond to those of the fluorescent modules, the FRET signal dynamics and the cAMP dependence are affected when the probe is fused to targeting domains. This limitation likely arises from the steric hindrance between the targeting domains that interfere with the conformational change required for the sensing mechanism [26,9].

In a recent study [9], this limitation was overcome by the development of a novel sensor for cAMP, named CUTie (for cAMP universal tag for imaging experiments), and engineered to minimize such interference. CUTie includes the CNBD B from the regulatory subunit type IIβ of PKA as the cAMP sensing moiety. Similarly to the earlier targeted Epac-based sensors, CFP is fused to the carboxyl terminus of the CNBD but the YFP moiety is removed from its amino-terminal end. Sequence conservation analyses and molecular simulations determined that the insertion of the YFP in an intra-domain loop of the CNBD (loop 4–5) was compatible with the correct folding of both the CNBD and the YFP. The advantage of this molecular architecture is that it leaves the amino-terminus of the CNBD free for fusion to arbitrary targeting domains, while the allosteric reactions remain confined to a hinging point near the lid of the cAMP binding pocket (Figure 1C). Contrary to the Epac sensor, CUTie produces a rise in FRET upon increasing cAMP concentrations.

When sensor localization was examined by expressing the targeted CUTie in neonatal rat ventricular myocytes, the probe was found to localize at the expected specific subcellular compartments (Figure 1D). Both the untargeted CUTie and its targeted versions were calibrated ‘in-cell’ to ascertain that they all respond with the same FRET change to a given amount of cAMP, an important feature as it allows quantitative assessment and direct comparison of cAMP levels at different subcellular sites [9]. When expressed in cardiac myocytes the CUTie reporters revealed that the cAMP response to β-ARs activation was significantly larger at the SR and SL compared with the bulk cytosol and the MF, resulting in an estimated twofold larger PKA activity at the SL and SR compared with the bulk cytosol and MF [9]. The varied local cAMP signals were found to be dependent on the activity of PDEs and signal heterogeneity was demonstrated to be necessary to achieve maximal efficiency of stimulated contraction and relaxation [9].

In the above study, targeting of the CUTie reporter was achieved by fusion to AKAP79, for localization at the SL, to AKAP18δ, for localization at the SR and to TPNI, for localization at the MF. The structural organization of ventricular cardiac myocytes is such that SL, SR, and MF come into very close proximity. The SL invaginates to form T tubules that are spaced ∼2 µm apart. Electron microscopy and super-resolution imaging studies show that the myofibrils form bundles of ∼0.6 µm in diameter and are tightly surrounded by SR cisternae [27]. Based on the architecture of these cells, the maximal distance between MF components and the SL can be estimated to be ∼1 µm and a maximal distance of only ∼300 nm separates sarcomere components and the SR. The fact that the targeted CUTie sensors detect distinct cAMP signals demonstrates that the size of the cAMP domains revealed by these probes is sub-microscopic. Mathematical modelling, supported by biochemical evidence, predicts that independent regulation of cAMP levels may occurs even at TPNI and MyBPC [9], two PKA targets that are spaced only ∼30 nm apart on the MF. The data thus indicate that the size of individual cAMP subcellular domains may be as small as the volume immediately surrounding functionally relevant cAMP targets. It is interesting to note that imaging of cAMP using non-targeted reporters fails to detect the same compartmentalized signals recorded by targeted sensors [28,10], an observation that raised in the past some skepticism on the notion of cAMP compartmentalization [29,30]. The recent findings indicate that detection of cAMP subcellular domains may require targeting of the sensor to the nanodomain. Probes that are free in the cytosol, although adequate to detect waves of cAMP propagation from the plasma membrane to the bulk cytosol are not capable of detecting nanodomain signals as the size of the subcellular domains where these occur is below the resolution limit of optical microscopy (200 nm). Unless the sensor is directly targeted to the domain, the local signal cannot be discerned as distinct from the signal in the bulk cytosol.

## Restricted propagation vs nanodomain regulation

**Figure 2.**
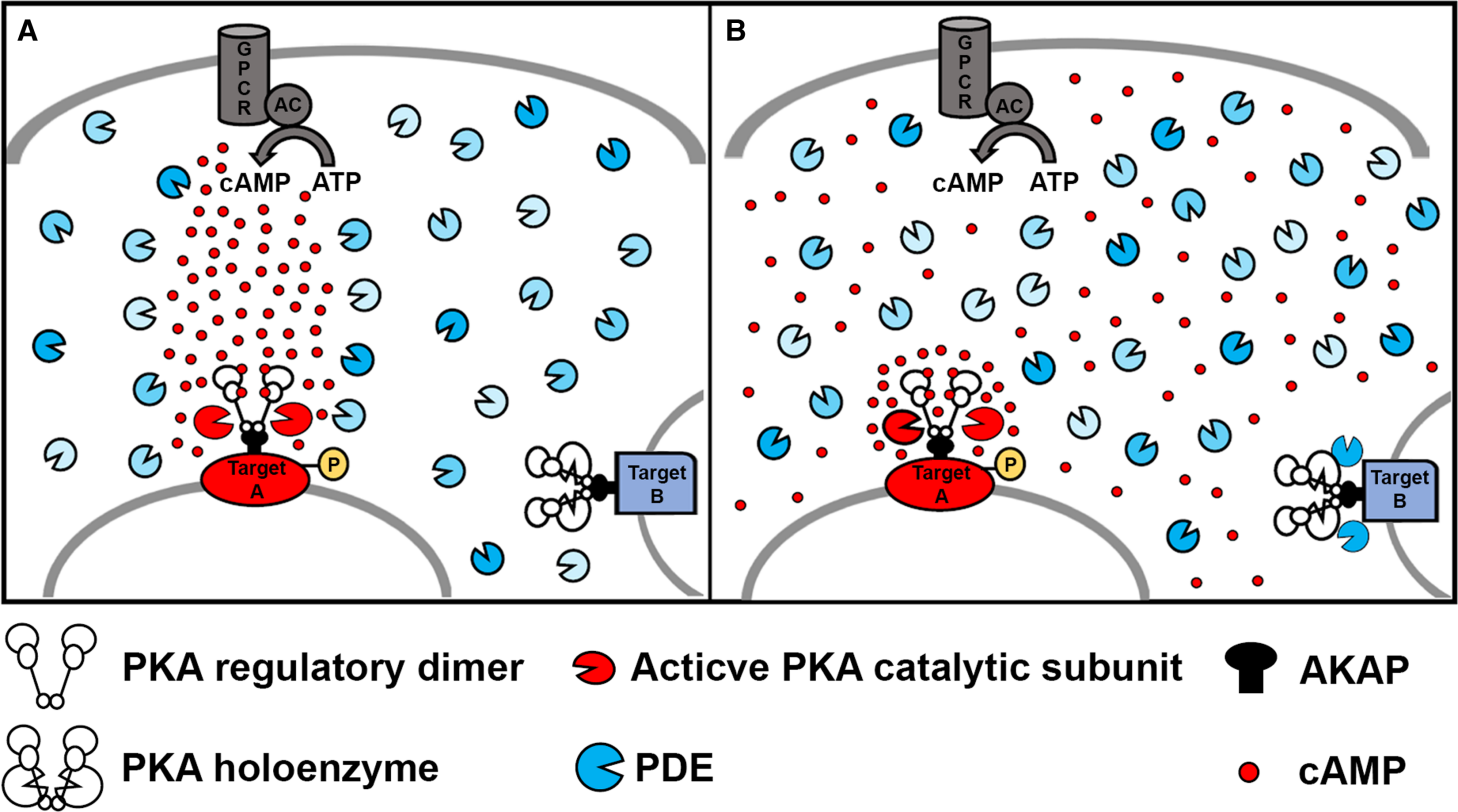
Restricted cAMP propagation vs nanodomain regulation. (**A**) Restricted propagation model. On activation of the Gs protein-coupled receptor (GPCR), the adenylyl cyclase (AC) synthetizes cAMP. The phospodiesterases (PDEs), by degrading cAMP, limit the diffusion of cAMP to the whole cells and restrict cAMP propagation from the focal point of synthesis at the plasma membrane to a downstream PKA target (target A), leading to its selective phosphorylation. (**B**) Nanodomain regulation model. cAMP freely propagates from the site of synthesis at the plasma membrane to the entire cell. PDEs anchored at specific subcellular sites act as a sink for cAMP, maintaining the level of the second messenger below the bulk cytosol and preventing activation of local PKA (exemplified by target B complex). At other sites, the absence of local PDEs and concomitant factors (see text for more details) allow for cAMP to accumulate locally at levels higher than bulk cytosol, leading to selective phosphorylation (target A). According to this model, the functional outcome of the cAMP signal is dictated by the local levels of the second messenger in the extremely restricted environment immediately surrounding specific effector/target protein complexes. PDE, shown here in different shades of blue to illustrate the diversity of the multiple isoforms, localize to specific subcellular sites via protein–lipid or protein–protein interactions (not shown for simplicity) which limit random diffusion of these enzymes.

Early studies on cAMP compartmentalization focused on the hormonal specificity of cAMP and how activation of different Gs-coupled receptors results in distinct cellular responses. These investigations showed that, for example, noradrenaline activates a subset of type II isoforms of PKA, whereas prostaglandin activates preferentially type I PKA. The two isoforms of PKA are localized via preferential anchoring to distinct AKAPs and selective activation of different PKA isoforms leads to phosphorylation of separate subsets of protein targets [31], explaining how cardiac inotropy is increased when cAMP levels rise in response to catecholamines, but not when the same amount of cAMP is generated on activation of the prostaglandin receptor [32]. Another example involves different isoforms of β-ARs: activation of β_1_-ARs and β_2_-ARs results in distinct physiological and pathophysiological responses. β_1_-ARs, but not β_2_-ARs, lead to phosphorylation of PLB and cardiac contractile proteins, hypertrophy, and apoptosis [33–35]. β_2_-AR localize exclusively to the deep T tubules and, using cytosolic FRET reporters and the local application of agonist, their activation has been shown to generate a local increase in cAMP. In contrast, β_1_-ARs are distributed across the entire cell surface and generate a more diffuse cAMP response throughout the cell. It has also become clear that different Gs-coupled receptors that localize to different regions on the plasma membrane can couple with distinct AC isoforms. ACs are uniquely regulated by calcium and other mechanisms and are organized in multiprotein complexes that may include AKAPs, PKA, PDEs, as well as PKC and PP2A [36–40]. As a consequence, ACs have been considered as central foci of cAMP compartmentalization [38] and often the problem of specificity of response has been described in terms of tunnelled propagation of cAMP from a focal point of synthesis at the plasma membrane to a defined subset of downstream targets. According to this view, a preferential coupling, or ‘communication’, exists between a specific receptor and a defined subcellular domain. Other parts of the cell do not experience the cAMP signal generated by activation of that receptor as they are protected via mechanisms that involve the action of PDEs (Figure 2A) [41,42].

However, the recent findings that the cAMP signal generated on activation of the β-AR is larger and faster at the PLB/SERCA complex on the SR and significantly smaller and delayed at the TPNI complex on the MF challenge this view and the model of ‘one receptor-one domain’ appears too simplistic. One interesting observation is that, in response to isoproterenol stimulation, the concentration of cAMP at specific sites can be higher (for example at AKAP79 on the SL or at AKAP18δ on the SR) or lower (at TPNI on MFs) than the concentration of the second messenger in the bulk cytosol. These findings shift the focus from a model of restricted propagation of cAMP from the site of synthesis to the bulk cytosol to one where the level of cAMP is locally regulated within individual nanodomains. According to this paradigm, cAMP can freely diffuse from the plasma membrane to the entire cell but the functional outcome of the cAMP signal is dictated by the local levels of the second messenger achieved in the extremely restricted environment immediately surrounding specific effector/target protein complexes (Figure 2B). At some specific sites, resident PDEs act as ‘sinks’ that locally degrade the second messenger to a level below that of the bulk cytosol and below the activation threshold of PKA, whereas at other sites cAMP levels can increase above bulk cAMP.

**Figure 3.**
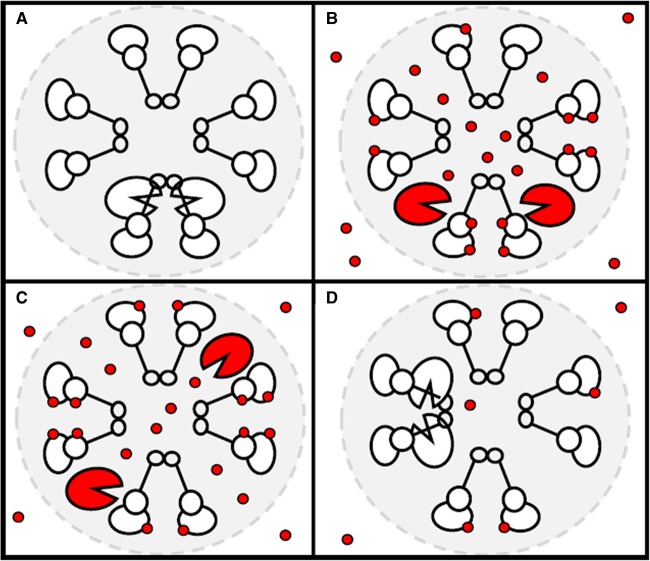
Hypothetical model for local ‘trapping’ of cAMP. (**A**) PKA holoenzyme and excess free PKA regulatory (R) subunits cluster at a specific subcellular site (or domain, depicted as a grey shaded area) where microviscosity may be higher than the surroundings. (**B**) When a cAMP signal is generated, cAMP diffuses from the site of synthesis at the plasma membrane and enters the domain where it binds to PKA holoenzyme to release active catalytic (C) subunits. In addition, cAMP also binds to free, excess R subunits localized within the domain. (**C**) When cAMP is released from the R subunit it can be readily re-captured by a free R subunits located nearby. cAMP is thus ‘trapped’ locally providing a higher cAMP concentration within the domain compared with the surrounding environment. (**D**) Eventually, cAMP diffuses out of the nanodomain and C subunits re-associate with R subunits locally. For an explanation of what each symbol represents see Figure 2.

Although experimental evidence shows that cAMP levels at some subcellular sites can increase above the level in the bulk cytosol [42,9] the mechanisms responsible remain elusive. One possibility, although speculative at this stage, is that this is achieved via a combination of low local PDE activity and a high concentration of cAMP binding proteins that provide a local buffer. For example, PKA regulatory (R) subunits have been reported to be expressed in several-fold excess of catalytic (C) subunits [43]. If free R subunits accumulate in excess at certain sites, cAMP could readily bind to them when the second messenger is released from another R subunit (Figure 3). This mechanism would prevent the dissipation of the local signal by ‘trapping’ cAMP within the domain. Local excess of free R subunit would also favour *in situ* re-association of C subunits. This would limit the distance from point of release over which C subunits can phosphorylate substrates, a mechanism that would further support compartmentalized phosphorylation of local targets [43]. Alternatively, intra-domain phosphorylation may rely on incomplete dissociation of C from R on PKA activation [44]. The above scenario could be favoured by the nanoscale dimension of the signalling domain, where the different molecular components may be present with approximately similar stoichiometry, and where the diffusivity of cAMP may be reduced due to increased local microviscosity [45].

## Novel physiological insight from nanodomain signalling

An unexpected finding using targeted versions of the CUTie reporter was that subcellular sites that contain PKA targets known to be activated by catecholamines in the fight-or-flight response, and therefore expected to belong to the same functional domain, in fact, experience cAMP signals of different amplitude and kinetics. As mentioned above, the data show that the cAMP signal detected in the environment surrounding the troponin complex at the MF is significantly smaller and delayed compared to the signal detected at the AKAP18δ/SERCA/PLB complex at the SR or the AKAP79/β-AR/AC/LTCC complex at the SL. Active PDEs are required for the signal to be compartmentalized, as the targeted sensors detect identical signals at the three sites when the PDEs are inhibited in the presence of isobutyl-metylxanthine, a non-selective PDEs inhibitor. Inhibition of PDEs, which results in a homogeneous increase in cAMP, also results in reduced myocyte shortening indicating that spatial coordination of distinct cAMP signals is required to achieve maximal efficiency of regulated contraction and relaxation. Why is the compartmentalized cAMP signal more effective than a uniform cAMP signal? The effect of PKA-dependent phosphorylation of LTCC and PLB is to enhance the Ca^2+^ transient and contraction, whereas PKA-mediated phosphorylation of TPNI reduces myofilament Ca^2+^ sensitivity and limits contraction. Therefore, a delayed and attenuated cAMP signal at the troponin complex may be necessary to fine tune the effect of catecholamines and avoid that the opposing effects of PKA on calcium handling counteract each other. Interestingly, PDE inhibitors used in the clinic for the treatment of heart failure lead to increased mortality in the long term. By obliterating the difference between cAMP nanodomains and by reducing the efficiency of contraction, non-selective PDE inhibition may curtail the benefit of local control of cAMP and a larger cAMP signal at TPNI may effectively deplete diastolic cardiac reserve via Ca^2+^ desensitization.

Although required to maximally enhance contraction and relaxation, attenuation of cAMP signals at the troponin complex comes with a downside. Experiments using animal models of cardiac hypertrophy and heart failure, which are characterized by down-regulation of the adrenergic system and reduced synthesis of cAMP, show that in hypertrophic/failing cardiac myocytes the cAMP signal at TPNI is dramatically reduced compared with the other sites, to a level that may not result in PKA activation. This may result in myofilament Ca^2+^ sensitization and diastolic dysfunction. In other words, the evidence suggests that the tighter regulation of cAMP levels at the troponin complex results in optimal enhancement of contraction and relaxation but this comes at the cost of intrinsic vulnerability of the myofilaments to Ca^2+^ sensitization, which may become apparent in conditions of reduced adrenergic drive.

These findings may also provide a rationale for the limited success of β-blocker therapy in heart failure with preserved ejection fraction (HFpEF), a condition that comprises almost half of the population burden of heart failure and for which there is currently no effective treatment [46]. HFpEF is a form of heart failure with normal left ventricular ejection fraction and where myocardial relaxation is impaired. While patients affected by heart failure with reduced ejection fraction unequivocally benefit from treatment with inhibitors of β-ARs, these drugs are not effective in HFpEF. One possible explanation could be that as in HFpEF the defect in myocyte function involves primarily relaxation, further reduction in TPNI phosphorylation and Ca^2+^ release from MFs in the presence of β-blockers would result in exacerbation of the functional deficit.

The novel insight provided by targeted reporters into the details of local cAMP signalling has important implications for the development of new therapeutic strategies [47,48]. For example, as the local cAMP domain at the TPNI complex is under the control of specific PDEs, identification of the PDE isoforms that selectively operate at this site would enable their specific targeting and may provide an effective means to re-establish the appropriate level of signalling at the MF by rectifying Ca^2+^ sensitization in pathological conditions. The possibility to treat disease with precision by targeting individual cAMP domains is exciting but an exhaustive molecular understanding of cAMP nanodomains localization, regulation and function is required before this potential can be exploited.

**Perspective**

- *Importance of the field*: It is now well established that cAMP mediates a plethora of functional effects while maintaining the fidelity of response via compartmentalization. FRET-based probes for cAMP, which can detect compartmentalized cAMP in intact cells with unprecedented accuracy, have been instrumental in defining the role of compartmentalized signalling. Recent developments using targeted probes to study cAMP signalling in cardiac myocytes are starting to provide novel mechanistic insight into cell physiology and its alterations in pathological conditions. These findings have important implication for the development of novel therapeutic strategies.
- *Summary of current thinking*: Recent evidence suggests that a delayed and attenuated cAMP signal at the troponin complex may be necessary to fine tune cardiac contraction and relaxation in response to catecholamines. Active PDEs are required for this compartmentalized signal since inhibition of PDEs results in a homogeneous increase in cAMP throughout the myocyte and reduced myocyte shortening. The current view is that the specific local control at these nanodomains is enabled by a distinct signalosome where effectors, targets, and regulators of the cAMP signal are clustered. Data show that the size of these domains can be as small as a few tens of nanometres, indicating that the functionally relevant cAMP signal is provided by the local concentration of cAMP in the space immediately surrounding the effector PKA within the signalosome, rather than by the global change in cAMP. At some sites, local PDEs act as sinks for cAMP, keeping the level of the second messenger below the bulk cytosol level. At other sites, cAMP raises above the bulk cytosol level through mechanisms that may involve low local PDE activity and high concentration of cAMP binding domains. This arrangement may prevent the dissipation of the local signal by ‘trapping’ cAMP within the nanodomain.
- *Future directions*: The current challenge in the field is to determine a comprehensive map of subcellular cAMP nanodomains and to understand the details of their molecular composition, regulation, and function. The recent findings in cardiac myocytes illustrate how an understanding of these molecular details may provide an opportunity to develop precision medicine approaches. The unique cAMP signalling occurring at the myofilament in response to sympathetic stimulation relies on local specific PDE isoforms. The identification of these enzymes will allow their selective targeting and may provide an effective means to re-constitute the appropriate level of cAMP signal in cardiac pathological conditions where calcium sensitization is affecting function. Further studies with targeted reporters will undoubtedly contribute to advance our understanding of the molecular basis of functional cAMP nanodomains, in the heart as well as in other organ systems.

## Abbreviations

β-ARs: β-adrenergic receptors
AC: adenylyl cyclase
AKAP: A-kinase anchoring protein
cAMP: 3′,5′-cyclic adenosine monophosphate
CFP: cyan fluorescent protein
CNBD: cyclic nucleotide-binding domain
CUTie: cAMP universal tag for imaging experiments
ECC: excitation–contraction coupling
Epac: exchange protein activated by cAMP
FRET: fluorescence resonance energy transfer
GPCRs: Gs protein-coupled receptors
HFpEF: heart failure with preserved ejection fraction
LTCC: L-type Ca^2+^ channels
MF: myofilaments
MyBPC: myosin-binding protein C
PDEs: phosphodiesterases
PKA: protein kinase A
PLB: phospholamban
PLM: phospholemman
PPs: phosphatases
RyRs: ryanodine receptors
SL: sarcolemma
SR: sarcoplasmic reticulum
TPNI: troponin I
YFP: yellow fluorescent protein
